# Teaching bioinformatics through the analysis of SARS-CoV-2: project-based training for computer science students

**DOI:** 10.1093/bioinformatics/btae208

**Published:** 2024-06-28

**Authors:** Pavlin G Poličar, Martin Špendl, Tomaž Curk, Blaž Zupan

**Affiliations:** Faculty of Computer and Information Science, University of Ljubljana, Večna pot 113, 1000 Ljubljana, Slovenia; Faculty of Computer and Information Science, University of Ljubljana, Večna pot 113, 1000 Ljubljana, Slovenia; Faculty of Computer and Information Science, University of Ljubljana, Večna pot 113, 1000 Ljubljana, Slovenia; Faculty of Computer and Information Science, University of Ljubljana, Večna pot 113, 1000 Ljubljana, Slovenia; Department of Education, Innovation and Technology, Baylor College of Medicine, 1 Baylor Plz, Houston, TX 77030, United States

## Abstract

**Motivation:**

We learn more effectively through experience and reflection than through passive reception of information. Bioinformatics offers an excellent opportunity for project-based learning. Molecular data are abundant and accessible in open repositories, and important concepts in biology can be rediscovered by reanalyzing the data.

**Results:**

In the manuscript, we report on five hands-on assignments we designed for master’s computer science students to train them in bioinformatics for genomics. These assignments are the cornerstones of our introductory bioinformatics course and are centered around the study of the severe acute respiratory syndrome coronavirus 2 (SARS-CoV-2). They assume no prior knowledge of molecular biology but do require programming skills. Through these assignments, students learn about genomes and genes, discover their composition and function, relate SARS-CoV-2 to other viruses, and learn about the body’s response to infection. Student evaluation of the assignments confirms their usefulness and value, their appropriate mastery-level difficulty, and their interesting and motivating storyline.

**Availability and Implementation:**

The course materials are freely available on GitHub at https://github.com/IB-ULFRI.

## 1 Introduction

While considerable attention has been devoted to structuring bioinformatics courses for life scientists (Mangul *et al.* 2017, Carey and Papin 2018, Madlung 2018), less attention has been paid to how to structure these courses for computer scientists (LeBlanc and Dyer 2004, Oesper and Vostinar 2020), especially those without a background in molecular biology. However, bioinformatics is an inherently interdisciplinary field and can be well approached from a computer science perspective. Skills in programming in Python or R, familiarity with databases, open access to information, and knowledge of data processing, visualization, and machine learning, provide not only an excellent entry point into bioinformatics (LeBlanc and Dyer 2004) but also wonderful opportunities for hands-on, project-based learning (Emery and Morgan 2017, Sauter *et al.* 2022). The role and benefits of project-based learning are well documented in the literature (Blumenfeld *et al.* 1991).

We propose a set of homework assignments and a corresponding syllabus for our Introduction to Bioinformatics course developed for master’s students in Computer Science. The course assumes a solid background in Python programming and is designed to introduce students with no prior knowledge of biology to the tools and analyses commonly performed in bioinformatics for genomics. In each of the five assignments presented here, we focus on a specific aspect of bioinformatics. We guide students through each topic through the theory and implementation of bioinformatics algorithms and their application to real-world data to solve practical problems. The construction of problem- and data-driven learning was also our main challenge in designing the problems in the exercises.

The assignments and the associated bioinformatics course we present here are designed to simulate the process of exploring the SARS-CoV-2 virus. As this course was originally developed during the initial coronavirus disease 2019 (COVID-19) lockdowns of 2020, we felt that this would be a particularly motivating example, as students would gain hands-on experience working with a virus that was, at the time, disrupting everyday life. The course begins with the assumption that the students have no prior knowledge of the SARS-CoV-2 virus, its structure, or its inner workings. Throughout the assignments, students progressively uncover different properties of SARS-CoV-2, which can then be validated against published scientific findings. In essence, students take on the role of scientists, immersing themselves in the discovery process to gain a deeper understanding of the virus over the course of the semester.

Below, we first present the didactic approach in designing the assignments. We then detail each of the five assignments, their associated learning objectives, and the problems that students will need to solve. We also discuss possible extensions and bonus problems for each assignment. Our proposed material was implemented at the Faculty of Computer and Information Science at the University of Ljubljana and evaluated by master’s students enrolled in the course. We present the results of this evaluation in a separate section. We conclude the manuscript with an overview of the achieved goals, providing information on the availability of the assignment text, code, and related resources.

## 2 Didactic approach

**Figure 1.**
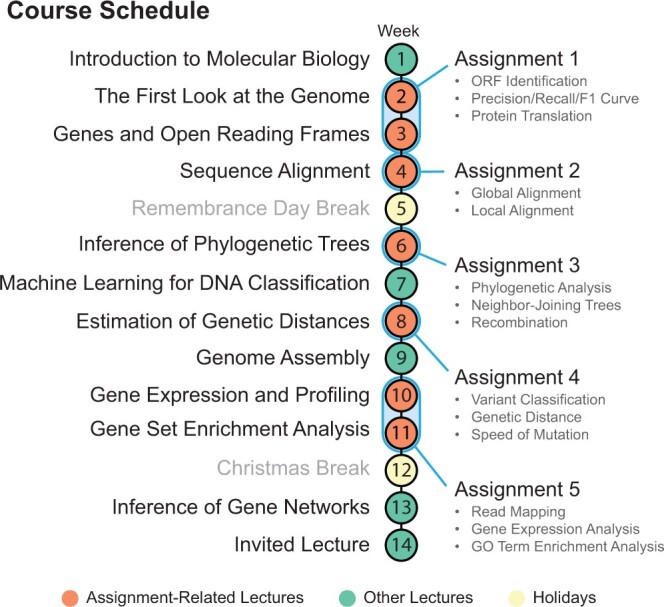
The course schedule for the winter semester of 2023–24 iteration of the Introduction to Bioinformatics course. Each numbered dot represents 1 week, with its associated lecture on the left. Lectures crucial to the completion of the corresponding homework assignment are indicated with red-colored dots and associated with the assignment with a blue outline.

The assignments and the associated bioinformatics course were originally developed during the 2020 COVID-19 lockdowns when in-person lab work was made difficult and project-based homework assignments were preferred. The bioinformatics course we designed (Fig. 1) is delivered in five cycles of about 3 weeks each, where in each cycle, students attend two to three lectures to learn theoretical concepts, followed by a practical homework assignment that reinforces the learned material. Each assignment focuses on a particular aspect of the bioinformatics workflow, implementing and applying these algorithms to real-world problems related to the SARS-CoV-2 pandemic. The text of the assignments is released in the middle of each cycle so that students can observe the problem they will have to solve, and the instructor can refer to the material during lectures. This interweaving of lectures and labs ensures that the students have a solid understanding of both the theory and applications of the presented bioinformatics algorithms.

The main objective of the course is to familiarize computer science students with the key concepts of molecular biology and bioinformatics. The lectures cover motivation and theory, and the homework assignments offer a practical opportunity for students to cement their knowledge by applying their skills in programming and data science algorithms. To assess the achievement of the learning outcomes, we administer a final examination at the end of the semester. While the focus of this manuscript is to describe the developed assignments, we are pleased to report that student pass rates have been excellent in all iterations of the course. We largely attribute this to the knowledge gained through practical work and the proposed project-based learning (For more information on the course structure and link to the Moodle page, see the instructor notes available at https://github.com/IB-ULFRI/instructor-notes.).

Each assignment consists of about four mandatory problems that follow our main investigative storyline of the SARS-CoV-2 virus, as well as optional problems that allow students to earn bonus points. These optional problems are meant to complement the mandatory problems and typically require additional analysis that serves as a point of interest or an alternative use or extension of a particular algorithm. For each assignment, students are required to submit a Python script containing their implementations of the required algorithms and a Jupyter Notebook report describing the steps, results, and interpretation of their analysis. Assignments are submitted through GitHub Classroom, an online classroom platform that allows instructors to create and manage assignments, distribute them to students, and receive submissions through GitHub. In their solutions, students were encouraged to use standard Python libraries for data access, analysis, and visualization, including biopython, pandas, matplotlib, and seaborn.

Project-based learning and coding to solve problems in bioinformatics primarily address the upper levels of Bloom’s Taxonomy (Bloom *et al.* 1956)—a framework for categorizing learning outcomes into levels of cognitive complexity—which involve higher-order thinking skills such as applying, analyzing, evaluating, and creating. Implementing algorithms in Python and applying them to practical problems of SARS-CoV-2 virus analysis facilitates the application of theoretical knowledge to real-world scenarios. As students immerse themselves in the discovery process, they are likely to evaluate the effectiveness of different algorithms and methods in uncovering information about the virus. Through the assignments, students synthesize different pieces of information and techniques to uncover known scientific facts about the virus. This creative process of simulating real scientific discovery requires a higher level of cognitive processing because students are not just learning existing knowledge, they are discovering it from the data.

## 3 Course assignments

The proposed course consists of five assignments that gradually introduce both molecular biology and fundamental concepts of bioinformatics to students of computer science.

### 3.1 Assignment 1: a first look at the genome

In the first assignment, students are familiarized with the basic concepts of genomics, including the roles of DNA and RNA, the differences between nucleic acids and amino acids, and the conceptual and functional grouping of some regions of DNA into *genes*. The problem set guides students through the process of finding and accessing genomic data from publicly available repositories, locating and filtering open reading frames (ORFs), and applying a naive classification scheme to identify ORFs corresponding to putative transmembrane proteins.

#### 3.1.1 Learning outcomes

In this assignment, students will:

- Acquaint with the biopython library to retrieve genomic records from the NCBI database and manipulate these records for further analysis.
- Implement an ORF finding algorithm and use it on a obtained nucleotide sequence.
- Examine the results to distinguish likely candidate ORFs from noise.

#### 3.1.2 Assignment tasks

In this assignment, we will be working with two different organisms: SARS-CoV-2 and *Escherichia coli* (*E. coli*). Our primary goal will be to develop an algorithm for identifying ORFs. However, using our developed algorithm without proper validation cannot give us confidence in our results. Therefore, we will first validate our approach to the well-annotated *E. coli* genome. Once we have confirmed that our procedure produces reasonable results, we can then apply it to the SARS-CoV-2 genome.

Before tackling the assignment, students must first install the biopython library and use it to download the NCBI *E. coli* record. The SARS-CoV-2 genome is provided in a separate FASTA file. The assignment comprises four problems:

1. Implement an ORF finding algorithm and apply it to the *E. coli* genome.
2. Using a permutation test, determine a filtering threshold to remove short ORFs likely appearing at random. Evaluate the reasonableness of this threshold by examining its precision, recall, and F1 metrics. We can compute these metrics by obtaining the ground truth genes from the NCBI *E. coli* record.
3. Having verified that the permutation test produces a reasonable threshold, apply the same treatment to the SARS-CoV-2 genome. Find all ORFs on the SARS-CoV-2 genome, and, using a permutation test, filter them down to only the most likely gene candidates.
4. Having identified the ORFs that likely correspond to true genes, we next implement a simple classification scheme to identify putative transmembrane proteins. Transmembrane proteins typically comprise a larger proportion of hydrophobic amino acids than non-transmembrane proteins. Therefore, by comparing the average hydrophobicity of the proteins corresponding to the ORF candidates against a background distribution of proteins from a known, annotated organism, we can infer likely transmembrane proteins. Translate each ORF identified in the third problem into its amino acid sequence and compute its average hydrophobicity. Using the hydrophobicity values of true proteins from *E. coli*, determine which of these SARS-CoV-2ORFs is most likely to correspond to transmembrane proteins.

#### 3.1.3 Bonus problems

At this stage, students can also create a visualization of the identified ORFs, showing their positions on the viral genome and marking which ORFs are on the positive and negative strands. They can determine whether SARS-CoV-2 is a large virus or not by downloading the metadata of all viruses in the NCBI virus database and comparing the lengths of the viral genomes. Alternatively, they can consider only RNA viruses.

#### 3.1.4 Summary

The first assignment guides students through the process of downloading, reading, and parsing genetic records. By implementing a simple ORF finding algorithm, students learn to work with genetic sequences and learn the importance of validating their algorithms against previously published findings. The aim of this assignment is to identify potential genes in the SARS-CoV-2 genome and hint that we can computationally determine their function, providing additional motivation for the second assignment.

**Figure 2.**
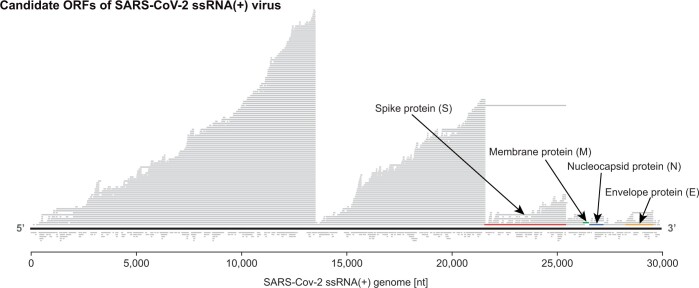
The ORFs identified by our naive ORF finding approach in the SARS-CoV-2 genome. Note that using our naive approach, we are able to recover only 10/12 functional ORFs due to frame-shift on ORF1ab (Naqvi *et al.* 2020). We highlight four of the identified ORFs corresponding to structural SARS-CoV-2 proteins.

A particularly interesting result from this assignment comes from the bonus problem in which students construct a visualization of their identified ORFs on the SARS-CoV-2genome shown in Fig. 2. From this plot, students observe almost no ORFs on the negative strand, a consequence of the single-stranded nature of the SARS-CoV-2virus.

### 3.2 Assignment 2: decoding gene function

In the first assignment, we identified ORFs corresponding to potential gene candidates in the SARS-CoV-2 virus. However, the nucleotide sequences alone provided little insight into the function of the potential downstream proteins. In this assignment, we develop a BLAST-like tool for the functional annotation of genes based on homologous genes. Using the Needleman–Wunsch algorithm for global alignment (Needleman and Wunsch 1970), we first identify closely related viruses and compile a database of annotated reference gene sequences. Then, using the Smith–Waterman algorithm for local alignment (Smith and Waterman 1981), we examine a selection of promising ORFs identified in the first assignment, verifying their correspondence to true genes and determining their protein function.

#### 3.2.1 Learning outcomes

In this assignment, students will:

- Examine the concept of homologous genes to infer gene function in related organisms.
- Implement and apply the Needleman–Wunsch algorithm for global alignment.
- Implement and apply the Smith–Waterman algorithm for local alignment.

#### 3.2.2 Assignment tasks

We provide students with 20 NCBI accession codes of related viruses from the *Coronaviridae* family, of which SARS-CoV-2 is a prominent member. We will first identify the most closely related viruses, and then use their annotated genes to determine the gene functions of a handful of ORFs we found in the previous homework assignment. The assignment comprises four problems:

1. Implement the Needleman–Wunsch algorithm for global alignment.
2. From the 20 provided sequences of related viruses, use your implementation of global alignment to find the three most closely related to SARS-CoV-2.
3. Implement the Smith–Waterman algorithm for local alignment.
4. In the first homework assignment, we identified several ORFs from SARS-CoV-2, which likely correspond to true genes. We will attempt to determine the function of five of these ORFs using local sequence alignment. Using the three most closely related viruses you identified in the second problem, compile a database of the true, reference genes along with their name and function from their annotated NCBI record. Use your implementation of local alignment to find the best matching reference gene in your database for each of the five ORFs from SARS-CoV-2. Determine the quality of the match and report your predicted protein function.

#### 3.2.3 Bonus problems

Our analysis reveals that the SARS-CoV-2 virus is closely related to the SARS-CoV virus, which caused the SARS outbreak between 2002 and 2004. In 2019, Xia *et al.* (2019) proposed a broad-spectrum human coronavirus inhibitory drug for the treatment of SARS. The drug works by binding to a specific motif of consecutive amino acid types in the spike protein of the coronaviruses, preventing binding and entry to human cells, and thereby blocking infection. Since the SARS-CoV-2 virus is closely related to SARS-CoV, could the same treatment work on SARS-CoV-2? Use local alignment and adapt the scoring matrix to identify potential medication target binding sites in the SARS-CoV-2 spike protein.

#### 3.2.4 Summary

In this assignment, students implement two sequence alignment algorithms, learn to identify related organisms, and infer protein functions from similar sequences of related organisms. We provide students with five ORFs identified in the first exercise, one of which does not correspond to an actual gene. By planting a false ORF, students need to reason about the results of the alignment procedures and identify the false ORF. The bonus problem shows an alternate use of the alignment algorithms and demonstrates that, by designing clever scoring functions, alignment algorithms can also be used for more complex tasks.

### 3.3 Assignment 3: mapping the family tree

In the second assignment, we used global alignment to determine the similarities between viruses to facilitate the functional annotation of putative genes. In this assignment, we will use these alignments and the neighbor-joining algorithm (Saitou and Nei 1987) to construct a phylogenetic tree of the *Coronaviridae* family of viruses. Using this phylogenetic tree, students explore and hypothesize about the evolutionary path of SARS-CoV-2. We also introduce the notion of recombination and investigate the role it may have played in the evolution of SARS-CoV-2.

#### 3.3.1 Learning outcomes

In this assignment, students will:

- Implement the neighbor-joining algorithm.
- Implement a drawing function for plotting phylogenetic trees.
- Construct a phylogeny for provided viral sequences and evaluate the importance of selecting an appropriate outgroup.
- Investigate potential recombination events.

#### 3.3.2 Assignment tasks

In this exercise, we provide students with 30 coronaviruses and one other viral sequence. The nucleotide sequences are pre-aligned using multiple-sequence alignment. The assignment comprises four problems:

1. Implement the neighbor-joining algorithm (Saitou and Nei 1987).
2. Implement a drawing function for plotting the resulting phylogenetic trees.
3. Using your implemented algorithms, construct and plot the phylogenetic tree of the 31 viral sequences. First, align the sequences using global alignment. Use the Hamming distance to calculate pairwise distances between the aligned sequences. Infer a phylogenetic tree and reroot it using the provided rerooting algorithm, using the unrelated Breda virus as the outgroup. The *Coronaviridae* family comprises four subgroups, which are contained within the NCBI records. Color each viral sequence according to its membership in each of these four subgroups.
4. Following the study from Lam *et al.* (2020), investigate the potential recombination event between the bat and pangolin viruses. Using their sliding window approach, determine whether particular regions of SARS-CoV-2 are more similar to the pangolin virus than the bat virus.

#### 3.3.3 Bonus problems

When constructing our phylogenetic trees, we inferred the single, most likely tree for our data. However, we do not know how robust this tree is and whether certain tree structures arise due to chance or reflect some true, underlying phenomenon. To estimate our uncertainty, we perform bootstrapping to assess the reliability of our trees. Implement phylogenetic tree bootstrapping and rerun your analysis. When drawing the resulting phylogenetic trees, we implemented standard, horizontal dendrograms. However, dendrograms come in all different shapes and sizes. Implement tree drawing using radial dendrograms as described in Bachmaier *et al.* (2005).

#### 3.3.4 Summary

**Figure 3.**
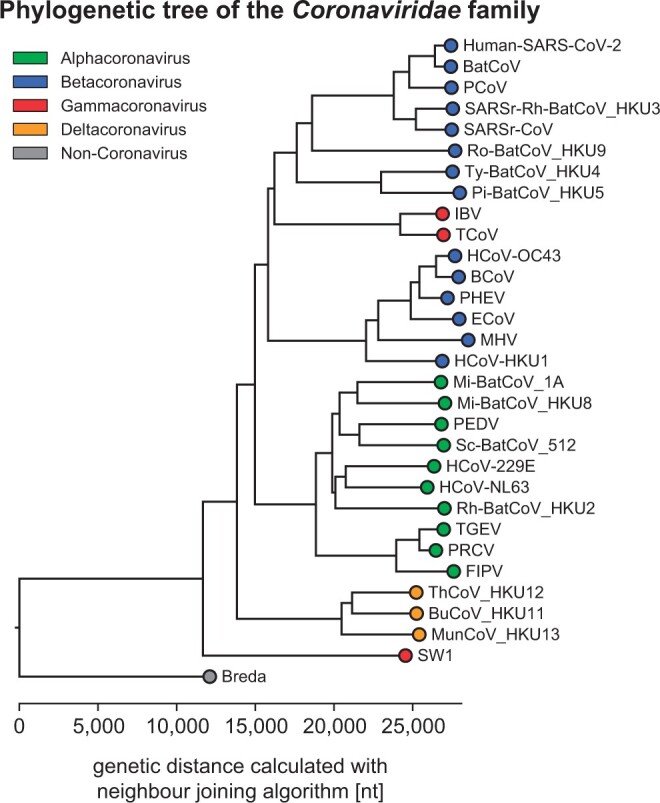
A phylogenetic tree of the *Coronaviridae* family of viruses obtained using the neighbor-joining algorithm. Leaves are colored according to their subgroup membership.

In this assignment, students use the neighbor-joining algorithm to construct a phylogenetic tree of the *Coronaviridae* family of viruses. Although the procedure produces an unrooted tree, we root the final tree to facilitate tree drawing. To compute distances between viral sequences, students use their implementation of global alignment from the previous assignment. Figure 3 shows the resulting dendrogram of this assignment. Upon closer inspection, students can observe that the dendrogram branches closely correspond to the four major subgroups of the *Coronaviridae* family.

### 3.4 Assignment 4: tracking viral evolution

In the third assignment, we considered viral evolution at a macro-level, mapping the evolutionary tree of the *Coronaviridae* family. In this assignment, we narrow our focus and investigate mutations within the SARS-CoV-2 virus species itself. Due to the unprecedented global response to the SARS-CoV-2 pandemic, timestamped SARS-CoV-2 sequences are abundant, allowing us to track the virus’s mutations through time in remarkable detail. The goal of the students in this assignment is 3-fold. First, to estimate the speed of mutation of the SARS-CoV-2 virus and compare it to the speed of mutation in other viruses. Secondly, learn about the differences between synonymous and non-synonymous mutations and their implications. Lastly, students must categorize different SARS-CoV-2 sequences into distinct viral variants based on each viral sequence’s observed single nucleotide variants. This classification allows us to analyze and plot the prevalence of these variants through time, offering a dynamic view of the virus’s evolution.

#### 3.4.1 Learning outcomes

In this assignment, students will:

- Calculate the speed of mutation and evaluate the impact of correction procedures like the Jukes–Cantor and the Kimura two-parameter correction models on mutation rate estimates.
- Differentiate between synonymous and non-synonymous mutations to assess their implications on genetic variation and evolutionary pressure.
- Classify viral sequences into variants.

#### 3.4.2 Assignment tasks

We provide students with 212 timestamped SARS-CoV-2 nucleotide and protein sequences gathered in Slovenia from the period between 2019 and 2022. Each sequence is pre-aligned to the NCBI reference sequence from 2019. Similarly, we provide pre-aligned nucleotide sequences for the unrelated Ebola and Zika viruses. Additionally, we provide variant classifications for a handful of Alpha and Delta variant SARS-CoV-2 sequences. The assignment comprises four problems:

1. Based on the NCBI reference sequence, calculate the number of mutations for each of the given SARS-CoV-2sequences and apply the Jukes–Cantor correction (Jukes and Cantor 1969). Plot the estimated number of mutations as a function of time. Determine the slope using linear regression and discuss how this relates to the speed of mutation.
2. Determine whether SARS-CoV-2 mutates quickly or slowly. To answer this question, repeat the same procedure as above on the Ebola and Zika viral sequences and compare the speed of mutation between the three viruses.
3. As the virus evolves, it accumulates mutations, and variants emerge. Compare the mutations on the sequences of Alpha and Delta viral variants. Plot the nucleotide mutation rates for four genes and identify the most common mutations. Determine if any mutations are shared between the two variants and look for evidence showing that the Delta variant evolved from the Alpha variant.
4. Determine which variant each of the 211 SARS-CoV-2 sequences belongs to. Develop a classification scheme to categorize viral protein sequences into variants based on the presence or absence of particular mutations (The mutations and their associated variants are collected from https://covariants.org). Then, plot the timeline of the emergence and prevalence of different SARS-CoV-2 variants in Slovenia throughout the COVID-19 pandemic.

#### 3.4.3 Bonus problems

Students may additionally implement the Kimura two-parameter correction model (Kimura 1980) and observe the changes in the analysis results if we instead use this model for genetic distance correction.

#### 3.4.4 Summary

**Figure 4.**
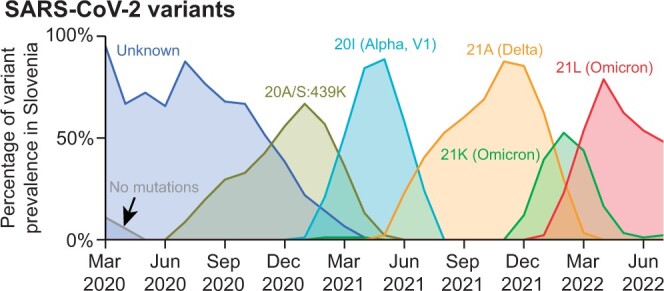
Emergence of variants in Slovenia between March 2020 and June 2022. Variants were sampled from the total sequences and smoothed with a moving average over 3 months.

In this assignment, students learn about viral mutation and the emergence of variants. They learn to estimate the speed of mutation and to compare these speeds among viruses. The difference between the speed of mutation at the nucleotide level and the genome level is of particular importance. The SARS-CoV-2 genome spans 30kbp and is longer than the other two Ebola and Zika virus genomes spanning 18 and 10 kbp, respectively. Comparing mutation rates at the genome level suggests that SARS-CoV-2 mutates faster than Ebola and Zika. However, a per-nucleotide comparison reveals that, in fact, the speed at which SARS-CoV-2 mutates is comparable to Ebola and is actually slower than Zika. Students also learn to categorize viral sequences into variants as defined by the broader scientific community. Students then construct a timeline of the prevalence of different variants in Slovenia, which clearly shows the emergence and decline of different variants (see Fig. 4).

### 3.5 Assignment 5: the immune response

So far, we have primarily concerned ourselves with the intrinsic aspects of the SARS-CoV-2 virus itself, including its structure, origins, and evolutionary path. However, it is important to recognize that viruses cannot function autonomously and require host cells to replicate. Therefore, any investigation of the SARS-CoV-2 virus is incomplete without also considering its interaction and influence on its host cells.

To investigate the effects of SARS-CoV-2 on the human body, we now turn our attention to human gene expression data. By examining the differences in the gene expression between healthy and infected human cells, we can determine which cellular processes are disrupted by the virus and reason about its implications for the host cells. The problem set guides students through a standard single-cell RNA-seq analysis involving the construction of count matrices, data normalization, differential expression analysis, and gene enrichment analysis. This approach provides a more comprehensive picture of the inner workings of the SARS-CoV-2 virus and reveals its broader biological impact on the human body.

#### 3.5.1 Learning outcomes

In this assignment, students will:

- Implement the construction of gene expression matrices.
- Execute a standard gene expression data analysis pipeline by performing dimensionality reduction, conducting clustering, and visualizing data patterns.
- Appraise the results by identifying differential expressions, and analyzing enriched Gene Ontology (GO) terms to elucidate biological significance.

#### 3.5.2 Assignment tasks

This assignment comprises two sections. In the first section, students learn to construct gene expression count matrices from synthetic reads using the algorithms developed in Assignment 2. In the second section, we conduct a full-fledged single-cell RNA-seq analysis on real-world data, characterizing the effects of SARS-CoV-2 infection on the human body.

**Section 1**. We provide students with 605 noisy, synthetic short reads corresponding to different chunks of the SARS-CoV-2 genome in FASTQ format and gene annotations for the SARS-CoV-2 genome in GFF format. The count matrix construction then comprises four steps:

1. From each read, extract the barcode and mRNA fragment.
2. Using local alignment, align each mRNA fragment to the SARS-CoV-2 genome.
3. If the fragment aligns to a region corresponding to a gene, update the matrix entry corresponding to the gene and associated cell barcode.
4. Apply basic matrix filtering based on the number of detected mRNA fragments for each cell and gene.

**Section 2**. In this section, we conduct a typical single-cell RNA-seq analysis using real-world data characterizing the human immune response to SARS-CoV-2 infection (Wilk *et al.* 2020). The data contain single cells obtained from healthy and infected donors. Additionally, we provide a subset of GO terms along with their associated genes.

The analysis then follows four steps:

1. Report the number of genes detected in each cell as well as the number of cells each gene was detected in. Based on their distributions, determine filtering thresholds for cells and genes.
2. Perform counts-per-million library-size normalization to account for sequencing depth and log normalization for variance stabilization.
3. Identify genes that are differentially expressed between healthy and infected donors. Perform comparisons between genes using the *t*-test followed by the Benjamini–Hochberg false discovery rate (FDR) correction to account for multiple comparisons. Compute gene log-fold changes and plot your results in a volcano plot.
4. Having obtained a list of differentially expressed genes, we next perform gene enrichment analysis, which may help us make sense of the genetic programs activated by the infection. Based on the list of differentially expressed genes, use the hypergeometric test to identify enriched GO terms. Inspect the identified GO terms and determine their relevance to the SARS-CoV-2 infection.

#### 3.5.3 Bonus problems

The differential expression analysis we performed above is one of the most common types of tasks in gene expression data analysis. However, gene expression data can be used in a myriad of other ways. For instance, one common task is the characterization of different cell types. These kinds of analyses typically involve dimensionality reduction, clustering, and visualization. In this exercise, we will use the scanpy Python library (Wolf *et al.* 2018). This exercise walks through these four different steps:

1. Run principal component analysis (Jolliffe 2002) on the gene expression matrix and extract the top 50 principal components. Visualize the first two components in a scatter plot.
2. Identify characteristic subpopulations of cells using a graph-based clustering algorithm of your choice.
3. Use t-distributed stochastic neighbor embedding (t-SNE) (Van der Maaten and Hinton 2008) or Uniform Manifold Approximation and Projection (UMAP) (McInnes *et al.* 2018) to construct a visualization of the data. Color the data points based on their cluster membership.
4. As before, perform differential expression analysis, this time finding differences between the different clusters of cells. Create a scatter plot of the t-SNE/UMAP embedding, this time coloring points according to the expression levels of the most highly differentially expressed genes.

#### 3.5.4 Summary

In the final exercise, we examine the impact of SARS-CoV-2 on the human body through the lens of gene expression data. First, students learn to construct gene expression matrices from synthetic reads, giving them a thorough understanding of this data modality. We then move on to a realistic example, where students follow the typical steps in a single-cell RNA-seq analysis. Through their analysis, students observe that infected individuals have a heightened immune response compared to their healthy counterparts. By experimenting with the protocol parameters, students discover that minor changes in the analysis parameters can result in different analysis results and findings. This underscores the importance of critically evaluating computational findings and the need for experimental validation.

## 4 Student evaluation

To evaluate the success of our course, we asked students to complete short, anonymous surveys after completing each of the five homework assignments. We report student participation rates in Table 1. We asked about each assignment’s interestingness, difficulty, time required to complete, clarity of instructions, and any suggestions for improvement for future homework assignments. Students also rated the complete set of assignments on these dimensions at the end of the course.

**Table 1.** We collected anonymous feedback from the 110 enrolled students attending the 2023/24 iteration of the course.^a^

HW1	HW2	HW3	HW4	HW5	Final
67	47	45	40	34	34

a We here report the number of students who provided feedback for surveys corresponding to each of the five homework assignments, as well as the final course feedback survey.

**Figure 5.**
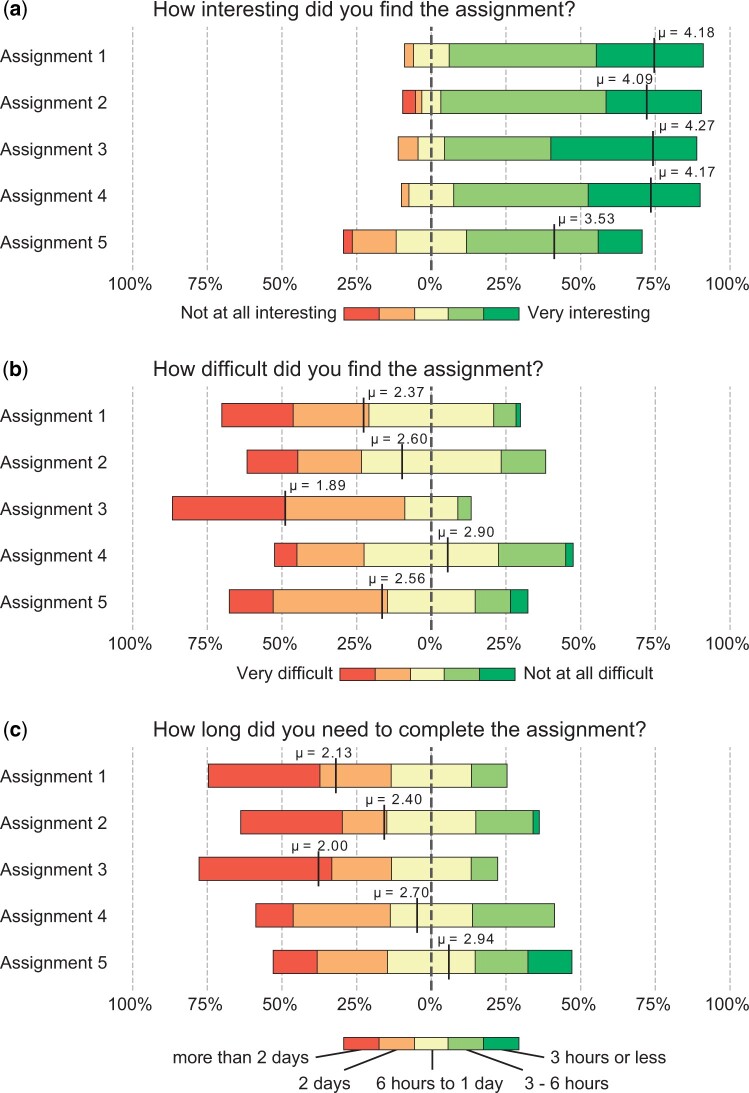
Student feedback. We plot the distributions of student responses related to assignment interestingness, difficulty, and time spent to complete the assignment. Each distribution is centered to the neutral response. To facilitate comparisons between the different assignments, we mark the distribution means, which are obtained by assigning 1 to the red option and 5 to the green option, and the intermediate options accordingly.

Figure 5 summarizes student feedback. For each of the three reported plots, we use the Kruskal–Wallis test to determine if there are significant differences between the independent groups, followed by Dunn’s post hoc test with FDR correction for multiple comparisons to identify specific pairs of groups with statistically significant differences in their ratings. Below, we report only those differences where p<0.05.

Figure 5a shows that students overwhelmingly found the assignments interesting. This confirms the many positive comments we received in the unstructured part of the feedback surveys. Of the assignments, students found the last assignment the least interesting. These ratings are largely consistent with the final rankings assigned to each assignment at the end of the course, where students assigned higher rankings to Assignments 4 and 3 with average final rankings of 3.8 and 3.5, respectively, and lower rankings to Assignments 1, 2, and 5 with average final rankings of 2.7, 2.7, and 2.3, respectively. Interestingly, students rated Assignments 1 and 2 highly upon completion but ranked them lower than Assignments 3 and 4 at the end of the course.

Figure 5b shows that, on average, students found the problems somewhat challenging. The difficulty of the problems may correlate with the algorithmic complexity of the tasks. For example, students rated Assignment 3 as the most difficult. This assignment requires students to implement the neighbor-joining algorithm, which is perhaps the most algorithmically challenging of the programming tasks. Several students reported struggling with its implementation in the unstructured feedback. Conversely, the fourth assignment, which requires very little algorithmic programming, was rated as easier than Assignments 1 and 3. These ratings are largely consistent with the final rankings assigned to each assignment at the end of the course, where students ranked Assignment 4 as the easiest with an average final ranking 2.4, Assignments 1, 2, and 5 as similarly difficult with average final rankings 3, 2.8, and 2.9, respectively, and Assignment 3 as the most difficult of all with an average final ranking of 3.9.

Our Introduction to Bioinformatics course is allocated six European Credit Transfer and Accumulation System credits, which corresponds to 150 working hours per semester. Divided among the five assignments with roughly 6 hours of accompanying lectures, each assignment should take about 24 working hours to complete. Figure 5c shows that most students completed the assignments within two full days, indicating that the assignments are roughly in line with this target. Assignment 1 took longer than tasks 2 and 3. Oddly, Assignment 5 typically took less time than the other assignments. This may be explained by our particular course logistics, where students only need to accumulate enough points to pass the course and do not need to complete all the assignments. Going into the final assignment, many students had likely already gathered sufficient points from previous assignments and invested less time.

We note here that the clarity of the instructions undoubtedly also affects the interestingness, difficulty, and time required to complete the tasks. For example, instruction clarity was positively correlated with interestingness (Spearman correlation ρ=0.34) and negatively correlated with time to complete (ρ=0.15). Interestingly, although the instruction clarity also negatively affected task difficulty (ρ=0.12), its *P*-value of 0.06 did not meet our significance threshold. These results suggest that although the clarity of instructions is essential for student engagement, it does not appear to have a significant effect on assignment difficulty.

In the unstructured section of the surveys, students shared overwhelmingly positive feedback, highlighting how much they enjoyed the structure and real-world nature of the assignments. They praised the engaging nature of the storyline, in which each assignment connects to the previous ones. Two students wrote: “I liked the guided building of the homework and the story that came with each task,” and “It feels like it’s actual work that could be done by scientists working on the field.” Some students commented on the applied nature of the assignments: “The guided building of the task and doing something that produced real-world results,” “The story behind what we were doing, didn’t feel like pointless algorithms,” and “Comparing real life examples together to give a feeling of actually working on something ‘real’.”

Students’ criticisms centered on the clarity of the assignment instructions and the lack of unit tests for the programming assignments. For example, “Maybe more examples/tests for the functions we have to write. Some of the exercise descriptions could have been clearer.” Since we have been collecting student feedback for all four iterations of the course and are constantly working to improve the assignments, we find it encouraging that the number of students who cited the instructions as a source of confusion decreases year over year. While we were initially reluctant to provide students with comprehensive unit tests to verify the correctness of their algorithms, assuming that their absence would encourage students to think about how to verify their implementations on their own, in practice, we now believe that providing unit tests is an overall benefit to students. Moving forward, we will likely provide accompanying unit tests in future installments of the course, allowing students to verify their implementations before moving on to the application exercises.

## 5 Conclusion

Project-based learning can be a great joy for both teachers and students. Here, we proposed a series of assignments in which computer science students learn about molecular biology through the implementation and application of bioinformatics algorithms. The assignments were designed to lead students through the discovery of the structure and function of the SARS-CoV-2virus, where the teacher’s role was to introduce the algorithms, while students used them to find genes and their functions, reason about the origin of the virus, and think about the human immune response triggered by the presence of the virus. The tasks were designed as breadcrumbs that lead students through the story of SARS-CoV-2, allowing them to piece together the intricate puzzle of life’s mechanisms and apply their computational skills to real-world biological challenges.

We, the authors, with much help from our students, have spent over 3 years adapting and refining the assignments proposed above. Every year, we collect student evaluations and opinions on each of the five assignments. Based on this feedback, we have been able to improve both the assignments and related lectures. According to the student evaluations, we have done well: students find the assignments interesting, engaging, and—to the delight of us instructors—sufficiently challenging. However, one of our greatest successes remained almost hidden: through project-based training and data-driven problem-solving, students learn about the world, the importance of data, apply critical thinking, and behave like true scientists. Next to training bioinformaticians, this is our most important achievement.

## Data Availability

We provide free and open access to all materials reported in the manuscript, including instructor notes, assignment instructions, and related data. These can all be found on GitHub at https://github.com/IB-ULFRI. For each assignment, we provide template repositories, which can be used in conjunction with the GitHub Classroom platform for course implementation. We can share assignment solutions privately with colleague instructors upon request.
