# Correction to: Teaching bioinformatics through the analysis of SARS-CoV-2: project-based training for computer science students

**DOI:** 10.1093/bioinformatics/btae635

**Published:** 2024-10-27

This is a correction to: Pavlin G Poličar, Martin Špendl, Tomaž Curk, Blaž Zupan, Teaching bioinformatics through the analysis of SARS-CoV-2: project-based training for computer science students, *Bioinformatics*, Volume 40, Issue Supplement_1, July 2024, Pages i20–i29, https://doi.org/10.1093/bioinformatics/btae208

In the originally published version of this manuscript, there was an error in the numbering of the section headings. The Student Evaluation section should be section 4 and the Conclusion should be section 5.

This has been corrected in the article.

